# 2-(4-Bromo­phen­yl)-5,6-methyl­enedi­oxy-3-phenyl­sulfinyl-1-benzofuran benzene solvate

**DOI:** 10.1107/S1600536809037283

**Published:** 2009-09-19

**Authors:** Hong Dae Choi, Pil Ja Seo, Byeng Wha Son, Uk Lee

**Affiliations:** a Department of Chemistry, Dongeui University, San 24 Kaya-dong Busanjin-gu, Busan 614-714, Republic of Korea; b Department of Chemistry, Pukyong National University, 599-1 Daeyeon 3-dong, Nam-gu, Busan 608-737, Republic of Korea

## Abstract

In the title compound, C_21_H_13_BrO_4_S·C_6_H_6_, the O atom and the phenyl group of the phenyl­sulfinyl substituent are located on opposite sides of the mean plane of the 5,6-methyl­enedioxy­benzofuran fragment; the phenyl ring is almost perpendicular to this plane [83.66 (6)°]. The 4-bromo­phenyl ring is rotated slightly out of the 5,6-methyl­enedioxy­benzo­furan plane, making a dihedral angle of 2.9 (1)°. The crystal structure is stabilized by inter­molecular C—H⋯O hydrogen bonds and inter­molecular C—H⋯π inter­actions. The crystal structure also exhibits π–π inter­actions between the benzene ring and the 4-bromo­phenyl ring of an adjacent mol­ecule [centroid–centroid distance = 3.586 (3) Å].

## Related literature

For the crystal structures of similar 5,6-methyl­enedi­oxy-1-benzofuran derivatives, see: Choi *et al.* (2007 ▶, 2008 ▶). For the pharmacological activity of benzofuran compounds, see: Howlett *et al.* (1999 ▶); Twyman & Allsop (1999 ▶). For natural products with benzofuran rings, see: Akgul & Anil (2003 ▶); von Reuss & König (2004 ▶).
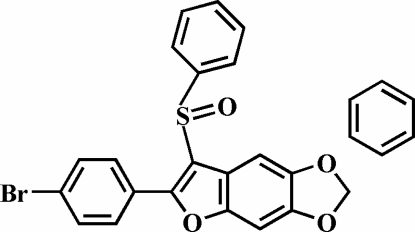


## Experimental

### 

#### Crystal data

C_21_H_13_BrO_4_S·C_6_H_6_
*M* *_r_* = 519.39
Monoclinic, 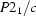

*a* = 13.8967 (7) Å
*b* = 12.6640 (7) Å
*c* = 13.0469 (7) Å
β = 102.418 (1)°
*V* = 2242.4 (2) Å^3^
*Z* = 4
Mo *K*α radiation
μ = 1.96 mm^−1^
*T* = 173 K
0.40 × 0.20 × 0.20 mm

#### Data collection

Bruker SMART CCD diffractometer
Absorption correction: multi-scan (*SADABS*; Sheldrick, 2000 ▶) *T*_min_ = 0.629, *T*_max_ = 0.679
13417 measured reflections
4886 independent reflections
3946 reflections with *I* > 2σ(*I*)
*R*_int_ = 0.075

#### Refinement

*R*[*F* ^2^ > 2σ(*F* ^2^)] = 0.036
*wR*(*F* ^2^) = 0.102
*S* = 1.02
4886 reflections
298 parameters
6 restraints
H-atom parameters constrained
Δρ_max_ = 0.64 e Å^−3^
Δρ_min_ = −0.65 e Å^−3^

### 

Data collection: *SMART* (Bruker, 2001 ▶); cell refinement: *SAINT* (Bruker, 2001 ▶); data reduction: *SAINT*; program(s) used to solve structure: *SHELXS97* (Sheldrick, 2008 ▶); program(s) used to refine structure: *SHELXL97* (Sheldrick, 2008 ▶); molecular graphics: *ORTEP-3* (Farrugia, 1997 ▶) and *DIAMOND* (Brandenburg, 1998 ▶); software used to prepare material for publication: *SHELXL97*.

## Supplementary Material

Crystal structure: contains datablocks global, I. DOI: 10.1107/S1600536809037283/kp2232sup1.cif

Structure factors: contains datablocks I. DOI: 10.1107/S1600536809037283/kp2232Isup2.hkl

Additional supplementary materials:  crystallographic information; 3D view; checkCIF report

## Figures and Tables

**Table 1:** Hydrogen-bond geometry (Å, °)

*D*—H⋯*A*	*D*—H	H⋯*A*	*D*⋯*A*	*D*—H⋯*A*
C4—H4⋯O4^i^	0.93	2.43	3.290 (3)	154
C14—H14⋯*Cg*1^ii^	0.93	2.72	3.531 (3)	147

Symmetry codes: (i) 
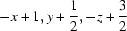
; (ii) 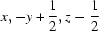. *Cg*1 is the centroid of the C1/C2/O1/C3/C9 furan ring.

**Fig. 1.**
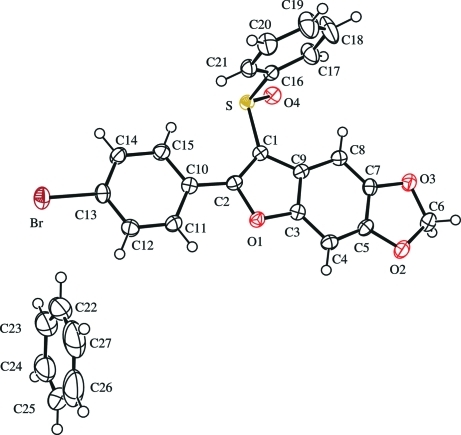
The molecular structure of the title compound with the atom numbering scheme. Displacement ellipsoids are drawn at the 50% probability level. H atoms are presented as a small cycles of arbitrary radius.

**Fig. 2.**
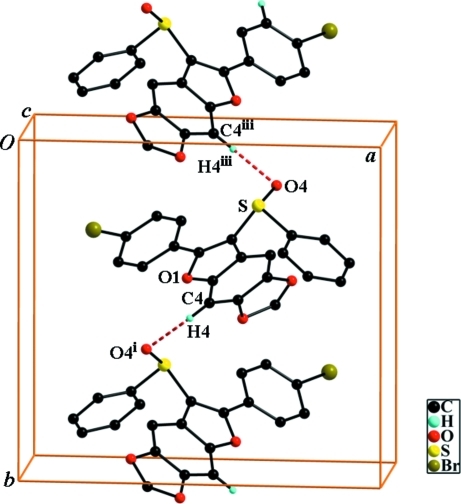
The C–H···O hydrogen bonds (dotted lines) in the crystal packing of (I). [Symmetry codes: (i) - x + 1, y + 0.5, - z + 3/2; (iii) - x + 1, y - 0.5, - z + 3/2.]

**Fig. 3.**
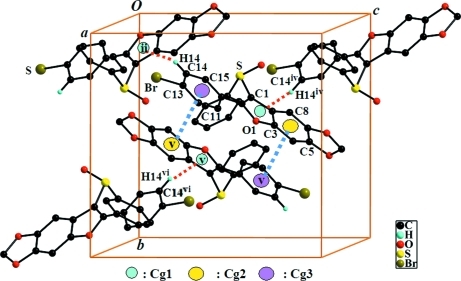
The C–H···π and π–π interactions (dotted lines) in (I). Cg denotes the ring centroids. [Symmetry codes: (ii) x, -y + 1/2, z - 1/2; (iv) x, - y + 1/2, z + 1/2; (v) - x + 1, - y + 1, - z + 1; (vi) - x + 1, y + 1/2, - z + 1/2.]

## supplementary crystallographic information

### Comment

Benzofuran systems have been received particular attention in the view of their pharmacological properties (Howlett *et al.*,  1999; Twyman & Allsop, 1999). These compounds occur in natural products (Akgul & Anil, 2003; von Reuss & König, 2004). As a part of our ongoing studies of the effect of side chain substituents on the solid state structures of 5,6-methylenedioxy-1-benzofuran analogues (Choi *et al.*, 2007, 2008) we report the crystal structure of the title compound (I) co-crystallized with benzene. The 5,6-methylenedioxybenzofuran unit is planar with a mean deviation of 0.030 (2) Å from the least-squares plane defined by the twelve constituent atoms. The dihedral angle formed by the planes of the 5,6-methylenedioxybenzofuran ring and 4-bromophenyl ring is 2.9 (1)°. The phenyl ring (C16-C21) is almost perpendicular to the plane of 5,6-methylenedioxybenzofuran [83.66 (6)°]. The crystal packing (Fig. 2) is stabilized by intermolecular C–H···O hydrogen bonds between a benzene H atom of the 5,6-methylenedioxybenzofuran ring and the oxygen of the S═O unit, with a C4–H4···O4^i^ (Table 1). The molecular packing (Fig. 3) is further stabilized by intermolecular C–H···π interactions between an H atom of the 4-bromophenyl ring and the furan ring, with a C14–H14···Cg1^ii^ (Table 1; Cg1 is the centroid of the C1/C2/O1/C3/C9 furan ring). The crystal structure (Fig. 3) also exhibits π–π interactions between the benzene ring and the 4-bromophenyl ring of the neighbouring molecules. The Cg2···Cg3^v^ distance is 3.586 (3) Å (Cg2 and Cg3 is the centroids of the C3/C4/C5/C7/C8/C9 benzene ring and the C10-C15 phenyl ring, respectively).

### Experimental

77% 3-Chloroperoxybenzoic acid (123 mg, 0.55 mmol) was added in small portions to a stirred solution of 2-(4-bromophenyl)-5, 6-methylenedioxy-3-phenylsulfanyl-1-benzofuran (213 mg, 0.5 mmol) in dichloromethane (30 mL) at 273 K. After being stirred at room temperature for 3 h, the mixture was washed with saturated sodium bicarbonate solution and the organic layer was separated, dried over magnesium sulfate, filtered and concentrated in vacuum. The residue was purified by column chromatography (hexane-ethyl acetate, 1:2 v/v) to afford the title compound as a colourless solid [yield 73%, m.p. 462-463 K; *R*_f_ = 0.71 (hexane-ethyl acetate, 1:2 v/v)]. Single crystals suitable for X-ray diffraction were prepared by slow evaporation of a solution of the title compound in benzene at room temperature.

### Refinement

All H atoms were positioned geometrically and refined using a riding model, with C–H = 0.93 Å for the aryl and 0.97 Å for the methylene H atoms. *U*_iso_(H) = 1.2*U*_eq_(C) for the aryl and the methylene H atoms. The distances of C–C in the solvated benzene ring were restrained to 1.39 (1) Å using command DFIX.

### Crystal data

**Table:**

C_21_H_13_BrO_4_S·C_6_H_6_	*F*(000) = 1056
*M**_r_* = 519.39	*D*_x_ = 1.538 Mg m^−^^3^
Monoclinic, *P*2_1_/*c*	Mo *K*α radiation, λ = 0.71073 Å
Hall symbol: -P 2ybc	Cell parameters from 6564 reflections
*a* = 13.8967 (7) Å	θ = 2.2–27.5°
*b* = 12.6640 (7) Å	µ = 1.96 mm^−^^1^
*c* = 13.0469 (7) Å	*T* = 173 K
β = 102.418 (1)°	Block, colorless
*V* = 2242.4 (2) Å^3^	0.40 × 0.20 × 0.20 mm
*Z* = 4	

### Data collection

**Table:**

Bruker SMART CCD diffractometer	4886 independent reflections
Radiation source: fine-focus sealed tube	3946 reflections with *I* > 2σ(*I*)
graphite	*R*_int_ = 0.075
Detector resolution: 10.0 pixels mm^-1^	θ_max_ = 27.0°, θ_min_ = 1.5°
φ and ω scans	*h* = −17→17
Absorption correction: multi-scan (*SADABS*; Sheldrick, 2000)	*k* = −16→8
*T*_min_ = 0.629, *T*_max_ = 0.679	*l* = −15→16
13417 measured reflections	

### Refinement

**Table:**

Refinement on *F*^2^	Primary atom site location: structure-invariant direct methods
Least-squares matrix: full	Secondary atom site location: difference Fourier map
*R*[*F*^2^ > 2σ(*F*^2^)] = 0.036	Hydrogen site location: difference Fourier map
*wR*(*F*^2^) = 0.102	H-atom parameters constrained
*S* = 1.02	*w* = 1/[σ^2^(*F*_o_^2^) + (0.0572*P*)^2^ + 0.3212*P*] where *P* = (*F*_o_^2^ + 2*F*_c_^2^)/3
4886 reflections	(Δ/σ)_max_ = 0.001
298 parameters	Δρ_max_ = 0.64 e Å^−^^3^
6 restraints	Δρ_min_ = −0.65 e Å^−^^3^

### Special details

**Table:**

Geometry. All esds (except the esd in the dihedral angle between two l.s. planes) are estimated using the full covariance matrix. The cell esds are taken into account individually in the estimation of esds in distances, angles and torsion angles; correlations between esds in cell parameters are only used when they are defined by crystal symmetry. An approximate (isotropic) treatment of cell esds is used for estimating esds involving l.s. planes.
Refinement. Refinement of F^2^ against ALL reflections. The weighted R-factor wR and goodness of fit S are based on F^2^, conventional R-factors R are based on F, with F set to zero for negative F^2^. The threshold expression of F^2^ > 2sigma(F^2^) is used only for calculating R-factors(gt) etc. and is not relevant to the choice of reflections for refinement. R-factors based on F^2^ are statistically about twice as large as those based on F, and R- factors based on ALL data will be even larger.

### Fractional atomic coordinates and isotropic or equivalent isotropic displacement parameters (Å^2^)

**Table:**

	*x*	*y*	*z*	*U*_iso_*/*U*_eq_	
Br	0.201403 (18)	0.26805 (2)	0.115664 (18)	0.03840 (10)	
S	0.63758 (4)	0.22232 (4)	0.56951 (4)	0.02291 (13)	
O1	0.43951 (10)	0.44110 (11)	0.59753 (11)	0.0246 (3)	
O2	0.57823 (12)	0.58271 (13)	0.94388 (13)	0.0359 (4)	
O3	0.71273 (11)	0.47027 (13)	0.96114 (12)	0.0311 (3)	
O4	0.68316 (11)	0.16954 (12)	0.67099 (11)	0.0302 (3)	
C1	0.56264 (14)	0.32535 (16)	0.60145 (15)	0.0224 (4)	
C2	0.47437 (14)	0.36076 (16)	0.54372 (16)	0.0226 (4)	
C3	0.50610 (14)	0.45414 (16)	0.69083 (16)	0.0230 (4)	
C4	0.49371 (15)	0.52551 (17)	0.76854 (17)	0.0267 (4)	
H4	0.4400	0.5708	0.7619	0.032*	
C5	0.56899 (16)	0.52172 (16)	0.85561 (17)	0.0261 (4)	
C6	0.66481 (17)	0.54604 (19)	1.01496 (17)	0.0324 (5)	
H6A	0.6472	0.5136	1.0757	0.039*	
H6B	0.7088	0.6048	1.0387	0.039*	
C7	0.64952 (15)	0.45403 (17)	0.86521 (16)	0.0250 (4)	
C8	0.66125 (15)	0.38315 (16)	0.78944 (16)	0.0245 (4)	
H8	0.7152	0.3381	0.7972	0.029*	
C9	0.58448 (15)	0.38419 (16)	0.69831 (16)	0.0227 (4)	
C10	0.41034 (15)	0.33635 (16)	0.44190 (16)	0.0234 (4)	
C11	0.32404 (16)	0.39505 (18)	0.40747 (18)	0.0296 (5)	
H11	0.3082	0.4482	0.4502	0.036*	
C12	0.26182 (16)	0.37585 (19)	0.31148 (18)	0.0319 (5)	
H12	0.2051	0.4160	0.2895	0.038*	
C13	0.28519 (16)	0.29598 (18)	0.24839 (17)	0.0277 (5)	
C14	0.36932 (17)	0.23670 (18)	0.28017 (18)	0.0300 (5)	
H14	0.3842	0.1833	0.2372	0.036*	
C15	0.43210 (17)	0.25665 (17)	0.37660 (19)	0.0291 (5)	
H15	0.4890	0.2166	0.3977	0.035*	
C16	0.73086 (15)	0.30455 (17)	0.53476 (16)	0.0243 (4)	
C17	0.81941 (17)	0.3206 (2)	0.60380 (19)	0.0423 (6)	
H17	0.8325	0.2886	0.6695	0.051*	
C18	0.8888 (2)	0.3853 (3)	0.5735 (2)	0.0584 (9)	
H18	0.9490	0.3971	0.6193	0.070*	
C19	0.8689 (2)	0.4326 (3)	0.4751 (2)	0.0530 (8)	
H19	0.9153	0.4772	0.4561	0.064*	
C20	0.78115 (18)	0.4138 (2)	0.40579 (19)	0.0381 (6)	
H20	0.7688	0.4443	0.3394	0.046*	
C21	0.71121 (16)	0.34943 (18)	0.43497 (16)	0.0284 (5)	
H21	0.6517	0.3363	0.3884	0.034*	
C22	0.0616 (2)	0.5412 (3)	0.1216 (2)	0.0578 (8)	
H22	0.1180	0.5166	0.1017	0.069*	
C23	−0.0017 (2)	0.4709 (3)	0.1512 (2)	0.0629 (9)	
H23	0.0124	0.3991	0.1516	0.076*	
C24	−0.0846 (2)	0.5040 (5)	0.1801 (3)	0.0906 (16)	
H24	−0.1278	0.4551	0.1987	0.109*	
C25	−0.1041 (3)	0.6067 (6)	0.1817 (4)	0.114 (2)	
H25	−0.1608	0.6279	0.2028	0.137*	
C26	−0.0441 (5)	0.6831 (4)	0.1536 (4)	0.129 (3)	
H26	−0.0592	0.7545	0.1557	0.155*	
C27	0.0434 (3)	0.6476 (4)	0.1207 (3)	0.0869 (13)	
H27	0.0860	0.6955	0.0995	0.104*	

### Atomic displacement parameters (Å^2^)

**Table:**

	*U*^11^	*U*^22^	*U*^33^	*U*^12^	*U*^13^	*U*^23^
Br	0.04220 (16)	0.04233 (17)	0.02635 (14)	−0.00182 (10)	−0.00222 (10)	−0.00305 (10)
S	0.0252 (3)	0.0199 (3)	0.0232 (3)	0.00213 (19)	0.0043 (2)	−0.0001 (2)
O1	0.0278 (7)	0.0209 (7)	0.0245 (7)	0.0025 (6)	0.0041 (6)	−0.0012 (6)
O2	0.0408 (9)	0.0339 (9)	0.0302 (8)	0.0055 (7)	0.0016 (7)	−0.0113 (7)
O3	0.0329 (8)	0.0324 (8)	0.0263 (8)	0.0004 (7)	0.0024 (6)	−0.0055 (7)
O4	0.0356 (8)	0.0263 (8)	0.0274 (8)	0.0057 (6)	0.0041 (6)	0.0055 (6)
C1	0.0247 (10)	0.0209 (10)	0.0223 (10)	0.0000 (8)	0.0065 (8)	0.0010 (8)
C2	0.0260 (10)	0.0184 (10)	0.0251 (10)	0.0003 (8)	0.0092 (8)	0.0006 (8)
C3	0.0261 (10)	0.0200 (10)	0.0229 (10)	−0.0009 (8)	0.0054 (8)	0.0027 (8)
C4	0.0286 (11)	0.0225 (11)	0.0299 (11)	0.0027 (8)	0.0083 (9)	−0.0001 (9)
C5	0.0338 (11)	0.0195 (10)	0.0263 (11)	−0.0016 (8)	0.0096 (9)	−0.0020 (8)
C6	0.0406 (12)	0.0290 (12)	0.0265 (11)	−0.0005 (10)	0.0047 (9)	−0.0036 (9)
C7	0.0279 (10)	0.0234 (10)	0.0232 (10)	−0.0047 (8)	0.0044 (8)	0.0008 (8)
C8	0.0258 (10)	0.0218 (10)	0.0265 (10)	0.0007 (8)	0.0072 (8)	0.0017 (9)
C9	0.0259 (10)	0.0195 (10)	0.0244 (10)	−0.0005 (8)	0.0090 (8)	0.0007 (8)
C10	0.0270 (10)	0.0199 (10)	0.0238 (10)	−0.0021 (8)	0.0064 (8)	0.0038 (8)
C11	0.0315 (11)	0.0254 (11)	0.0311 (11)	0.0044 (9)	0.0046 (9)	−0.0049 (9)
C12	0.0307 (11)	0.0292 (12)	0.0333 (12)	0.0064 (9)	0.0013 (9)	−0.0007 (10)
C13	0.0314 (11)	0.0269 (11)	0.0228 (10)	−0.0042 (9)	0.0015 (8)	0.0020 (9)
C14	0.0358 (12)	0.0266 (11)	0.0273 (11)	0.0034 (9)	0.0064 (9)	−0.0054 (9)
C15	0.0307 (11)	0.0276 (11)	0.0282 (11)	0.0062 (9)	0.0043 (9)	0.0006 (9)
C16	0.0246 (10)	0.0245 (10)	0.0245 (10)	−0.0007 (8)	0.0065 (8)	−0.0028 (9)
C17	0.0326 (12)	0.0649 (18)	0.0269 (12)	−0.0063 (12)	0.0007 (10)	0.0041 (12)
C18	0.0341 (14)	0.095 (3)	0.0395 (15)	−0.0256 (15)	−0.0062 (12)	0.0027 (16)
C19	0.0452 (15)	0.070 (2)	0.0439 (15)	−0.0286 (14)	0.0111 (12)	0.0008 (15)
C20	0.0414 (13)	0.0430 (15)	0.0304 (12)	−0.0073 (11)	0.0090 (10)	0.0042 (11)
C21	0.0284 (10)	0.0296 (11)	0.0256 (10)	0.0001 (9)	0.0022 (9)	−0.0010 (9)
C22	0.0416 (15)	0.085 (2)	0.0443 (16)	0.0091 (15)	0.0035 (13)	−0.0037 (16)
C23	0.0460 (16)	0.092 (3)	0.0480 (17)	0.0026 (17)	0.0039 (14)	−0.0090 (18)
C24	0.0445 (18)	0.177 (5)	0.0487 (19)	−0.003 (2)	0.0070 (15)	−0.038 (3)
C25	0.072 (3)	0.176 (6)	0.076 (3)	0.057 (3)	−0.025 (2)	−0.081 (4)
C26	0.148 (5)	0.089 (4)	0.102 (4)	0.060 (4)	−0.080 (4)	−0.049 (3)
C27	0.093 (3)	0.076 (3)	0.067 (2)	0.003 (2)	−0.036 (2)	0.000 (2)

### Geometric parameters (Å, °)

**Table:**

Br—C13	1.901 (2)	C12—H12	0.9300
S—O4	1.4968 (15)	C13—C14	1.376 (3)
S—C1	1.774 (2)	C14—C15	1.391 (3)
S—C16	1.795 (2)	C14—H14	0.9300
O1—C3	1.371 (2)	C15—H15	0.9300
O1—C2	1.382 (2)	C16—C17	1.375 (3)
O2—C5	1.370 (3)	C16—C21	1.393 (3)
O2—C6	1.429 (3)	C17—C18	1.387 (4)
O3—C7	1.381 (2)	C17—H17	0.9300
O3—C6	1.435 (3)	C18—C19	1.389 (4)
C1—C2	1.370 (3)	C18—H18	0.9300
C1—C9	1.442 (3)	C19—C20	1.374 (4)
C2—C10	1.464 (3)	C19—H19	0.9300
C3—C9	1.391 (3)	C20—C21	1.383 (3)
C3—C4	1.396 (3)	C20—H20	0.9300
C4—C5	1.370 (3)	C21—H21	0.9300
C4—H4	0.9300	C22—C23	1.365 (5)
C5—C7	1.394 (3)	C22—C27	1.370 (5)
C6—H6A	0.9700	C22—H22	0.9300
C6—H6B	0.9700	C23—C24	1.354 (4)
C7—C8	1.371 (3)	C23—H23	0.9300
C8—C9	1.416 (3)	C24—C25	1.330 (7)
C8—H8	0.9300	C24—H24	0.9300
C10—C15	1.395 (3)	C25—C26	1.377 (8)
C10—C11	1.400 (3)	C25—H25	0.9300
C11—C12	1.382 (3)	C26—C27	1.445 (8)
C11—H11	0.9300	C26—H26	0.9300
C12—C13	1.386 (3)	C27—H27	0.9300
			
O4—S—C1	106.19 (9)	C14—C13—C12	120.8 (2)
O4—S—C16	106.93 (9)	C14—C13—Br	119.17 (17)
C1—S—C16	97.18 (10)	C12—C13—Br	120.07 (17)
C3—O1—C2	107.24 (15)	C13—C14—C15	120.0 (2)
C5—O2—C6	106.02 (17)	C13—C14—H14	120.0
C7—O3—C6	105.75 (16)	C15—C14—H14	120.0
C2—C1—C9	107.91 (17)	C14—C15—C10	120.5 (2)
C2—C1—S	127.83 (16)	C14—C15—H15	119.8
C9—C1—S	124.22 (15)	C10—C15—H15	119.8
C1—C2—O1	109.40 (17)	C17—C16—C21	121.3 (2)
C1—C2—C10	136.67 (19)	C17—C16—S	120.88 (17)
O1—C2—C10	113.92 (17)	C21—C16—S	117.76 (16)
O1—C3—C9	110.75 (17)	C16—C17—C18	118.7 (2)
O1—C3—C4	123.88 (18)	C16—C17—H17	120.7
C9—C3—C4	125.33 (19)	C18—C17—H17	120.7
C5—C4—C3	112.83 (19)	C17—C18—C19	120.4 (2)
C5—C4—H4	123.6	C17—C18—H18	119.8
C3—C4—H4	123.6	C19—C18—H18	119.8
C4—C5—O2	126.47 (19)	C20—C19—C18	120.3 (2)
C4—C5—C7	123.47 (19)	C20—C19—H19	119.8
O2—C5—C7	110.06 (19)	C18—C19—H19	119.8
O2—C6—O3	108.32 (17)	C19—C20—C21	119.9 (2)
O2—C6—H6A	110.0	C19—C20—H20	120.1
O3—C6—H6A	110.0	C21—C20—H20	120.1
O2—C6—H6B	110.0	C20—C21—C16	119.3 (2)
O3—C6—H6B	110.0	C20—C21—H21	120.3
H6A—C6—H6B	108.4	C16—C21—H21	120.3
C8—C7—O3	126.87 (19)	C23—C22—C27	121.0 (4)
C8—C7—C5	123.75 (19)	C23—C22—H22	119.5
O3—C7—C5	109.38 (18)	C27—C22—H22	119.5
C7—C8—C9	114.48 (18)	C24—C23—C22	121.0 (4)
C7—C8—H8	122.8	C24—C23—H23	119.5
C9—C8—H8	122.8	C22—C23—H23	119.5
C3—C9—C8	120.13 (18)	C25—C24—C23	119.7 (5)
C3—C9—C1	104.69 (17)	C25—C24—H24	120.2
C8—C9—C1	135.18 (19)	C23—C24—H24	120.2
C15—C10—C11	118.1 (2)	C24—C25—C26	123.1 (4)
C15—C10—C2	122.52 (19)	C24—C25—H25	118.4
C11—C10—C2	119.36 (18)	C26—C25—H25	118.4
C12—C11—C10	121.5 (2)	C25—C26—C27	117.2 (4)
C12—C11—H11	119.2	C25—C26—H26	121.4
C10—C11—H11	119.2	C27—C26—H26	121.4
C11—C12—C13	119.1 (2)	C22—C27—C26	118.0 (5)
C11—C12—H12	120.5	C22—C27—H27	121.0
C13—C12—H12	120.5	C26—C27—H27	121.0
			
O4—S—C1—C2	−144.86 (18)	S—C1—C9—C3	−178.71 (15)
C16—S—C1—C2	105.13 (19)	C2—C1—C9—C8	178.1 (2)
O4—S—C1—C9	32.39 (19)	S—C1—C9—C8	0.4 (3)
C16—S—C1—C9	−77.62 (18)	C1—C2—C10—C15	1.9 (4)
C9—C1—C2—O1	1.4 (2)	O1—C2—C10—C15	−179.02 (18)
S—C1—C2—O1	179.01 (14)	C1—C2—C10—C11	−177.7 (2)
C9—C1—C2—C10	−179.5 (2)	O1—C2—C10—C11	1.4 (3)
S—C1—C2—C10	−1.9 (4)	C15—C10—C11—C12	−0.4 (3)
C3—O1—C2—C1	−1.2 (2)	C2—C10—C11—C12	179.2 (2)
C3—O1—C2—C10	179.43 (16)	C10—C11—C12—C13	0.6 (3)
C2—O1—C3—C9	0.6 (2)	C11—C12—C13—C14	−0.3 (3)
C2—O1—C3—C4	−177.09 (19)	C11—C12—C13—Br	−179.72 (17)
O1—C3—C4—C5	178.27 (18)	C12—C13—C14—C15	0.0 (3)
C9—C3—C4—C5	0.9 (3)	Br—C13—C14—C15	179.35 (17)
C3—C4—C5—O2	179.17 (19)	C13—C14—C15—C10	0.2 (3)
C3—C4—C5—C7	0.2 (3)	C11—C10—C15—C14	0.0 (3)
C6—O2—C5—C4	176.6 (2)	C2—C10—C15—C14	−179.6 (2)
C6—O2—C5—C7	−4.3 (2)	O4—S—C16—C17	−8.5 (2)
C5—O2—C6—O3	6.8 (2)	C1—S—C16—C17	100.9 (2)
C7—O3—C6—O2	−6.7 (2)	O4—S—C16—C21	170.16 (16)
C6—O3—C7—C8	−175.7 (2)	C1—S—C16—C21	−80.45 (18)
C6—O3—C7—C5	4.1 (2)	C21—C16—C17—C18	1.8 (4)
C4—C5—C7—C8	−1.0 (3)	S—C16—C17—C18	−179.6 (2)
O2—C5—C7—C8	179.93 (19)	C16—C17—C18—C19	−0.2 (5)
C4—C5—C7—O3	179.20 (19)	C17—C18—C19—C20	−1.5 (5)
O2—C5—C7—O3	0.1 (2)	C18—C19—C20—C21	1.5 (5)
O3—C7—C8—C9	−179.72 (18)	C19—C20—C21—C16	0.1 (4)
C5—C7—C8—C9	0.5 (3)	C17—C16—C21—C20	−1.8 (3)
O1—C3—C9—C8	−179.05 (17)	S—C16—C21—C20	179.57 (18)
C4—C3—C9—C8	−1.4 (3)	C27—C22—C23—C24	0.4 (5)
O1—C3—C9—C1	0.2 (2)	C22—C23—C24—C25	−1.3 (5)
C4—C3—C9—C1	177.88 (19)	C23—C24—C25—C26	1.0 (7)
C7—C8—C9—C3	0.6 (3)	C24—C25—C26—C27	0.3 (7)
C7—C8—C9—C1	−178.4 (2)	C23—C22—C27—C26	0.9 (5)
C2—C1—C9—C3	−1.0 (2)	C25—C26—C27—C22	−1.2 (6)

### Hydrogen-bond geometry (Å, °)

**Table:**

*D*—H···*A*	*D*—H	H···*A*	*D*···*A*	*D*—H···*A*
C4—H4···O4^i^	0.93	2.43	3.290 (3)	154
C14—H14···Cg1^ii^	0.93	2.72	3.531 (3)	147

Symmetry codes: (i) −*x*+1, *y*+1/2, −*z*+3/2; (ii) *x*, −*y*+1/2, *z*−1/2.
